# Genetic evidence links invasive monk parakeet populations in the United States to the international pet trade

**DOI:** 10.1186/1471-2148-8-217

**Published:** 2008-07-24

**Authors:** Michael A Russello, Michael L Avery, Timothy F Wright

**Affiliations:** 1 Department of Biology and Physical Geography, University of British Columbia Okanagan, Kelowna, British Columbia V1V 1V7, Canada; 2 Centre for Species at Risk and Habitat Studies, University of British Columbia Okanagan, Kelowna, British Columbia V1V 1V7, Canada; 3 USDA Wildlife Services, National Wildlife Research Center, Gainesville, Florida 32641, USA; 4 Department of Biology, New Mexico State University, Las Cruces, NM 88003, USA

## Abstract

**Background:**

Severe ecological and economic impacts caused by some invasive species make it imperative to understand the attributes that permit them to spread. A notorious crop pest across its native range in South America, the monk parakeet (*Myiopsitta monachus*) has become established on four other continents, including growing populations in the United States. As a critical first step to studying mechanisms of invasion success in this species, here we elucidated the geographical and taxonomic history of the North American invasions of the monk parakeet. Specifically, we conducted a genetic assessment of current monk parakeet taxonomy based on mitochondrial DNA control region sequences from 73 museum specimens. These data supported comparative analyses of mtDNA lineage diversity in the native and naturalized ranges of the monk parakeet and allowed for identification of putative source populations.

**Results:**

There was no molecular character support for the *M. m. calita*, *M. m. cotorra*, and *M. m. monachus *subspecies, while the Bolivian *M. m. luchsi *was monophyletic and diagnosably distinct. Three haplotypes sampled in the native range were detected within invasive populations in Florida, Connecticut, New Jersey and Rhode Island, the two most common of which were unique to *M. m. monachus *samples from eastern Argentina and bordering areas in Brazil and Uruguay.

**Conclusion:**

The lack of discrete morphological character differences in tandem with the results presented here suggest that *M. m. calita, M. m. cotorra *and *M. m. monachus *are in need of formal taxonomic revision. The genetic distinctiveness of *M. m. luchsi *is consistent with previous recommendations of allospecies status for this taxon. The geographic origins of haplotypes sampled in the four U.S. populations are concordant with trapping records from the mid-20th century and suggest that propagule pressure exerted by the international pet bird trade contributed to the establishment of invasive populations in the United States.

## Background

The introduction of exotic species into native ecosystems has modified habitats, reduced species diversity and adversely altered ecosystem functioning across the globe [1]. In the United States only habitat degradation poses a higher threat to endangered taxa [2]. In other regions around the world, however, as many as 80% of endangered species are threatened due to pressures from non-native species [3]. From an economic perspective, the environmental damage caused by the approximately 50,000 alien-invasive species in the United States, coupled with the costs of controlling these species, exceeds $120 billion per year [1]. The severe ecological and economic impacts of invasive species render it imperative to understand the attributes that permit them to establish and spread within their expanded ranges.

The monk parakeet (*Myiopsitta monachus*) is one of the most successful parrot invaders [4]. It has been historically regarded as an agricultural pest in its native range in South America, as noted by Charles Darwin during his voyage on the H.M.S. Beagle:

"A small green parrot (*Conurus murinus*; early synonym of *M. monachus*), with a grey breast, appears to prefer the tall trees on the islands to any other situation for its building-place. A number of nests are placed so close together as to form one great mass of sticks. These parrots always live in flocks, and commit great ravages on the corn-fields. I was told that near Colonia 2500 were killed in the course of one year." [[5], Chapter VII, p. 101]

Over the past century, the widespread introduction of *Eucalyptus *has facilitated the expansion of *M. monachus *populations in its native range [6-8]. In Argentina, this rapid increase in native population sizes has been implicated in the loss of 2–15% of sunflower and corn yields, crop damages estimated by some sources to be as high as US$1 billion per year [9,10]. In addition to rapid population growth within their endemic range, monk parakeets have become broadly established on four other continents, presumably due to their widespread presence in the international pet bird trade. Despite the rapid spread of monk parakeets around the globe and their potential as an agricultural pest, little is known about the geographical history of the invasions. Such information may provide important insights into the mechanisms of invasion success and potential for future range expansions.

**Figure 1:**
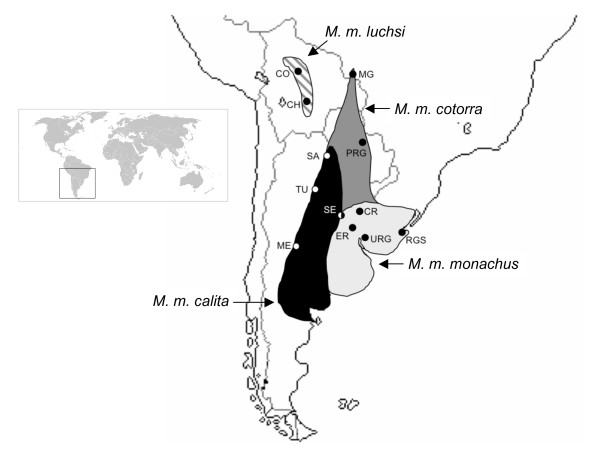
**Distribution of *Myiopsitta monachus *across its native range in South America.** Alternative shading denotes the individual ranges of the four subspecies [redrawn from [7]] including *M. m. monachus *(light gray), *M. m. calita *(black), *M. m. cotorra *(dark gray), and *M. m. luchsi *(striped). Localities of specimens sampled for this study are indicated by dots, with associated abbreviations following Table 1.

The monk parakeet is distributed in its native range across the lowlands of South America, east of the Andes from Bolivia to Patagonia [[11], Figure 1]. Four subspecies are currently recognized based on geographical variation in wing length, bill size, body mass and plumage coloration [12]. The nominate, *M. m. monachus*, is the largest of the four subspecies and is found in extreme southeast Brazil (Rio Grande do Sul), Uruguay and northeastern Argentina (provinces of Entre Rios, Santa Fe, Córdoba, south to northern Rio Negro). *M. m. calita *is distributed in western Argentina from Salta province south to Rio Negro and described as having bluer wings and a darker gray head. *M. m. cotorra *is distributed in southeast Bolivia (department of Tarija), Paraguay, southern Brazil (Mato Grosso do Sul), south to northern Argentina (provinces of Formosa and Chaco). *M. m. cotorra *has been reported as brighter green on the upper parts and less yellowish on the abdomen than *M. m. calita *[13], yet their general lack of distinctiveness in these characters and their similarity in size has brought their status as separate taxonomic entities into question [13]. Lastly, *M. m. luchsi *is geographically and altitudinally isolated from the other subspecies, restricted to the arid, intermontane valleys of the east Andes in Bolivia, from southern Cochabamba to northern Chuquisaca [11]. In addition, *M. m. luchsi *exhibits distinctive plumage coloration, reported as generally brighter than the other subspecies, with a bright yellow lower breast, paler underwings, a dark area at the base of the upper mandible, and a breast entirely pale grey without the barred effect observed in the other three subspecies [11]. In contrast to the colonial, tree-nesting behavior of all other monk parakeets, *M. m. luchsi *build single-chambered nests on cliffs. These behavioral and morphological differences led del Hoyo [14] to elevate this group to allospecies status (*Myiopsitta luchsi*), a designation that is not widely recognized.

In addition to the South American populations, naturalized breeding populations of *M. monachus *have been established in such disparate regions as the United Kingdom, Puerto Rico, Kenya, Japan, Spain, Italy, Belgium, Czech Republic, and throughout the United States including growing populations in Florida, Texas and Connecticut [4,15-18]. The origin of initial invaders in the U.S. has been traced back to purposeful and accidental releases of individuals from the pet trade for which approximately 64,225 monk parakeets were imported between 1968–1972 alone [4]. In general, U.S. naturalized populations are a collection of disjunct colonies, most common in southern and coastal regions, with an estimated 6,000 to 200,000 individuals in residence nationally [19]. Once feared as a potentially devastating crop pest, *M. monachus *is still generally considered a moderate threat as populations continue to grow exponentially [15,19]. A less publicized impact of the monk parakeet invasion has been their preference for power structures as nesting substrates. In 2001, an estimated 1,027 power outages in south Florida were attributed to monk parakeet activities at an approximate cost of $585,000 [20]. Moreover, the cost of nest removal alone in south Florida was estimated at $1.3 to $4.7 million over the past five years (2003–2007) [21]. In addition to the financial impacts to energy providers and the communities they serve, monk parakeets may have ecological effects within the local ecosystems. Overall, these real and potential impacts have resulted in the presence of statewide controls or bans in over 15 states [22].

The objectives of this study were to identify the taxonomic and geographic source(s) of the invasive populations along the eastern seaboard of the United States. As the accuracy of monk parakeet taxonomy has been questioned, we initially conducted a genetic assessment of the biological validity of the four currently recognized subspecies of *M. monachus *by way of historical DNA analysis from museum specimens collected throughout the native range. This broad sampling of mitochondrial DNA lineage diversity in the endemic range provides a reference database by which to infer the origin and extent of mtDNA haplotype diversity in invasive populations in Florida, Connecticut, New Jersey and Rhode Island. Our results suggest that these invasive populations are derived from a localized area in eastern Argentina and bordering areas in Brazil and Uruguay within the described range of *M. m. monachus*, the most commonly exported subspecies for the international pet trade.

## Methods

### Sampling

Toepad tissue was obtained from 73 museum specimens of *Myiopsitta monachus *representing all four subspecies (*M. m. calita*, n = 9; *M. m. cotorra*, n = 16; *M. m. luchsi*, n = 14; *M. m. monachus*, n = 38) courtesy of the American Museum of Natural History (AMNH). Blood samples from four individuals of *M. m. monachus *collected in Entre Rios, Argentina were also included in the sampling. In addition, feather or tissue samples were obtained from 64 individuals from four localities across the naturalized range of *M. monachus *in the eastern United States. Specifically, muscle tissue was obtained from individuals culled in Miami, Florida (n = 43) as part of a management program by the local electric utility company. Feathers were obtained from colonies sampled from different trees and areas in Bridgeport, Connecticut (n = 9) and Edgewater, New Jersey (n = 11). In addition, we sampled a single museum specimen collected in Kent County, Rhode Island (AMNH832643). Table 1 includes detailed collection information for all samples while Figure 1 plots the individual sampling localities within the native range of *M. monachus*.

**Table 1:** Sampling of *Myiopsitta monachus *subspecies in native and naturalized ranges

Subspecies	N	Country	Province/Department/State	Abbreviation	Accession #^†^
*M. m. calita*	3	Argentina	Santiago del Estero	SE	140653, 474808–474809
	1	Argentina	Mendoza	ME	147931
	5	Argentina	Tucumán	TU	474803–474807
*M. m. cotorra*	5	Brazil	Mato Grosso	MG	127356–127359, 474802
	11	Paraguay	Concepción	PRG	149404, 320771–320777, 748687–748688, 811356
*M. m. luchsi*	12	Bolivia	Chuquisaca	CH	139094–139096,139098–139106
	2	Bolivia	Cochabamba	CO	139107, 148194
*M. m. monachus*	2	Argentina	Santiago del Estero	SE	140649, 140651
	2	Argentina	Salta	SA	474796–474797
	14*	Argentina	Entre Rios	ER	779017–779019, 779025, 779037, 779059–779061, 779065, 779083
	13	Argentina	Corrientes	CR	793580–793581, 793586–793587, 793589, 793597–793598, 793603, 793605, 793616, 793631, 793633, 793640
	6	Brazil	Rio Grande do Sul	RGS	321247–321249, 321560–321562
	1	Uruguay	Río Negro	URG	474800
Unknown^††^	9	United States	Connecticut	CT	n/a
	43	United States	Florida	FL	n/a
	11	United States	New Jersey	NJ	n/a
	1	United States	Rhode Island	RI	832643

*Sampling includes four field-collected samples.

^† ^Accession numbers of specimens sampled in the collections at the American Museum of Natural History or, in the case of Florida individuals, at the USDA National Wildlife Research Center.

^†† ^Individuals sampled in the naturalized populations are of unknown taxonomic affinity.

### Data collection

DNA was extracted from blood, tissue, and feather samples using the DNeasy Tissue kit and manufacturer protocols (Qiagen, Inc.). Museum specimens were handled in a dedicated ancient DNA facility using a modified Qiagen DNeasy Tissue kit protocol [23]. Other necessary precautions were taken to prevent and detect contamination by contemporary specimens, including use of extraction and PCR negative controls, PCR amplification of short, overlapping fragments (see below), and confirmation of all unique haplotype sequences by way of cloning [24].

A 558 basepair segment of the mitochondrial DNA (mtDNA) control region (CR) was amplified as a single fragment using external primers LGlu and CR522Rb [25] for the DNA extractions from blood and feather samples or, in the case of the DNA extractions from museum specimens, as a set of four overlapping fragments not exceeding 180 basepairs in length each [Lglu/MyiopCR1B (TGCCAATGGTTGCCCTAATAA); MyiopCR2A (GACATTGCATGCTCGTCCTA)/MyiopCR2B (TGGAATTGGAGAGGAGTGTTTT); MyiopCR3A (AGCAACTAAACCGAATGATCC)/MyiopCR3B (TGGGCCTGAAGCTAGTAACG); MyiopCR4A (CCACTCACGAGAAACCATCA)/CR522Rb]. All PCR reactions were carried out on an MJ Research DNA Engine thermal cycler in 25 μl reactions containing: ~20–50 ng of DNA, 10 mM Tris-HCl (pH 8.3), 50 mM KCl, 1.5 mM MgCl_2_, 200 μM dNTPs, 0.5 μM of each primer and 0.5 U of AmpliTaq Gold DNA polymerase (Applied Biosystems). Cycling conditions for all primer pairs consisted of 95°C for 10 minutes, 35 cycles of 95°C for 30 seconds, 50°C for 30 seconds, 72°C for 30 seconds, and a final extension of 72°C for 7 minutes. Double-stranded PCR products were sequenced using Big Dye 3.1 terminators on an ABI 3730 DNA sequencer (Applied Biosystems).

### Population genetic analyses

Previous work revealed duplication and concerted evolution of the control region in *Amazona *and *Pionus *parrots [25]. Subsequent surveys of mtDNA gene order across the entire order of parrots via PCR across selected gene junctions has revealed that this duplication is absent in many parrot species, including *M. monachus *(Schirtzinger E, Gonzalez L, Eberhard JR, Graves G, Wright TF, unpublished data). Furthermore, long-range PCR followed by sequencing of the entire mtDNA genome of *M. monachus *has shown that it conforms to a typical avian gene order with a single control region (Schirtzinger E, Eberhard JR, Wright TF, unpublished data).

Haplotypic (h) [26] and nucleotide (π) [26] diversity estimates were calculated based on mtDNA CR sequences as executed in ARLEQUIN [27]. Pairwise genetic distances were calculated in PAUP*4.0b10 [28] assuming the HKY+G model of nucleotide substitution as selected according to the Akaike information criterion as implemented in Modeltest [29]. Levels of genetic divergence between samples were calculated with the fixation index (PhiST) [30] as executed in ARLEQUIN [27]. Because the HKY model is not implemented in ARLEQUIN the more inclusive Tamura-Nei (TrN) [31] model with the same parameters for ti/tv rate and α was used. Significance of PhiST for all possible pairwise population comparisons was assessed using 2,000 permutations. Tests for significant geographic structure among subspecies sampled across the native range were conducted using analysis of molecular variance (AMOVA) [30]. MtDNA CR sequence alignments for all four subspecies were further employed to identify diagnostic nucleotide sites by means of population aggregation analysis [32]. The presence of characters fixed within and differing among populations was used as evidence to diagnose distinct units.

### Network and phylogenetic analyses

Sequences were unambiguously aligned in Clustal X [33] employing default settings for gap opening and extension costs. Genealogical relationships among all sampled haplotypes throughout the native range were reconstructed as a haplotype network using the statistical parsimony method of Templeton et al. [34] as implemented in TCS, version 1.06 [35]. Gaps were treated as a 5^th ^character state. Networks are especially appropriate for inferring intraspecific gene genealogies because of the potential for extant ancestral nodes and multifurcating relationships [29].

A Bayesian haplotype tree was reconstructed using MrBayes 3.1 [36] assuming the HKY+G model of nucleotide substitution as selected by Modeltest [29] as described above. The orange-chinned parakeet (*Brotogeris jugularis*) was used as an outgroup to root the tree, as previous phylogenetic studies have revealed species from this genus to be sister to *M. monachus *[37,38]. The Bayesian phylogenetic analysis ran four simultaneous chains for 2.0 × 10^6 ^total generations, each using a random tree as a starting point, the default heating scheme, and saving a tree every 100 generations for a total 20,000 trees. The first 2,000 trees were discarded as burn-in samples and the remaining 18,000 trees were used to construct a majority-rule consensus tree and derive posterior probability values. Violation of a criterion of monophyly was used to indicate incorrect taxonomic assignment.

## Results

### Within subspecies variation

A total of 17 mtDNA CR haplotypes were recovered among the 77 individuals sampled from across the native range of the four described subspecies of *M. monachus *(GenBank Accession No. EU545521-EU545537). The number of haplotypes identified ranged from four (*M. m. calita*) to eight (*M. m. monachus*), with levels of haplotypic and nucleotide diversity relatively consistent across the subspecies (Table 2). Of the 17 detected haplotypes, three were shared among a combination of *M. m. calita*, *M. m. cotorra*, and *M. m. monachus*. One shared haplotype was widely distributed, sampled in individuals from all three of these subspecies in disparate localities ranging from northern (Tucumán province) and central (Entre Rios and Mendoza provinces) Argentina, to Concepción, Paraguay and Mato Grosso, Brazil. All five haplotypes recovered for *M. m. luchsi *in Bolivia were unique to that subspecies. Overall, sequence divergence among *M. monachus *haplotypes recovered from the four subspecies ranged from 0.20% to 1.66% (*luchsi01/calita02*) based on HKY+G distances.

**Table 2:** Genetic variation within *Myiopsitta monachus *subspecies

Subspecies	n	No. of	Haplotypic	Nucleotide
		Haplotypes^†^	Diversity, h	Diversity, π
*M. m. calita*	9	4	0.58	0.0031
			(0.18)^‡^	(0.0022)
*M. m. cotorra*	16	5	0.73	0.0020
			(0.079)	(0.0015)
*M. m. monachus*	38	8	0.77	0.0028
			(0.040)	(0.0019)
*M. m. luchsi*	14	5	0.66	0.0015
			(0.12)	(0.0013)
Unknown (U.S.A.)	64	4	0.52	0.0025
			(0.042)	(0.0017)

^†^Results based on 558 base pairs of the mtDNA control region. All haplotypes recovered for each subspecies are considered. Three haplotypes were shared among *M. m. calita*, *M. m. cotorra*, and *M. m. monachus*. All haplotypes sampled in U.S.A. were also recovered in native range (see text).

^‡^Values in parentheses are the standard errors for h and π.

### Among subspecies differentiation

Genetic variation across the samples was highly structured with significant levels of genetic variation distributed among, rather than within, the four *M. monachus *subspecies (p < 0.0001; Table 3a). When the Bolivian *M. m. luchsi *was removed from the AMOVA, the results were reversed, with the vast majority of variation distributed within (96.41%) rather than among (3.59) subspecies (Table 3b). A similar pattern was revealed by the fixation indices, with all pairwise comparisons involving *M. m. luchsi *highly significant (Table 3c). None of the pairwise comparisons of *M. m. calita*, *M. m. cotorra *and *M. m. monachus *approached significance. Likewise, *M. m. luchsi *was diagnosably distinct from each of the other three subspecies, with the number of diagnostic characters detected ranging from three (*M. m. cotorra*, *M. m. monachus*) to five (*M. m. calita*) across the 558 basepairs of the mtDNA CR (Table 3c).

**Table 3:** Genetic divergence among *Myiopsitta monachus *subspecies

a. Analysis of molecular variance including all subspecies				
Subspecies	Source of	d.f.	% of	P-value
	variation^‡^		variation	

*M. m. calita*	Among	3	61.23	<0.0001
*M. m. cotorra*	Within	74	38.77	
*M. m. monachus*	Total	77		
*M. m. luchsi*				
				
b. Analysis of molecular variance excluding *M. m. luchsi*				
Subspecies	Source of	d.f.	% of	P-value
	variation^‡^		variation	
*M. m. calita*	Among	2	3.59	0.1369
*M. m. cotorra*	Within	61	96.41	
*M. m. monachus*	Total	63		
				
c. Diagnostic characters and fixation indices*				
Subspecies	*M. m. calita*	*M. m. cotorra*	*M. m. monachus*	*M. m. luchsi*
*M. m. calita*	-	-0.0370	0.0500	0.8062**
*M. m. cotorra*	0	-	0.0422	0.8266**
*M. m. monachus*	0	0	-	0.7757**
*M. m. luchsi*	5	3	3	-

‡ Among populations, within populations or total.

* Number of diagnostic characters (below diagonal) and PhiST (above diagonal) based on mtDNA control region sequence data.

** Indicates statistical significance (p < 0.001).

### Genealogical relationships

**Figure 2:**
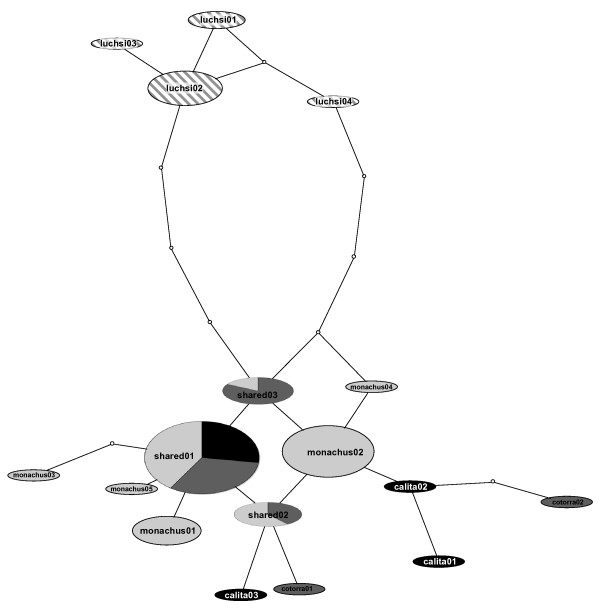
**Network showing genealogical relationships among *Myiopsitta monachus *haplotypes sampled in the native range.** Haplotypes are connected with a 95% confidence limit. The size of each oval is proportional to the frequency of the haplotype in the analysis. White dots represent mutational steps separating the observed haplotypes. Different shades represent the proportion of individuals of each subspecies exhibiting that particular haplotype (colors as in Figure 1).

**Figure 3:**
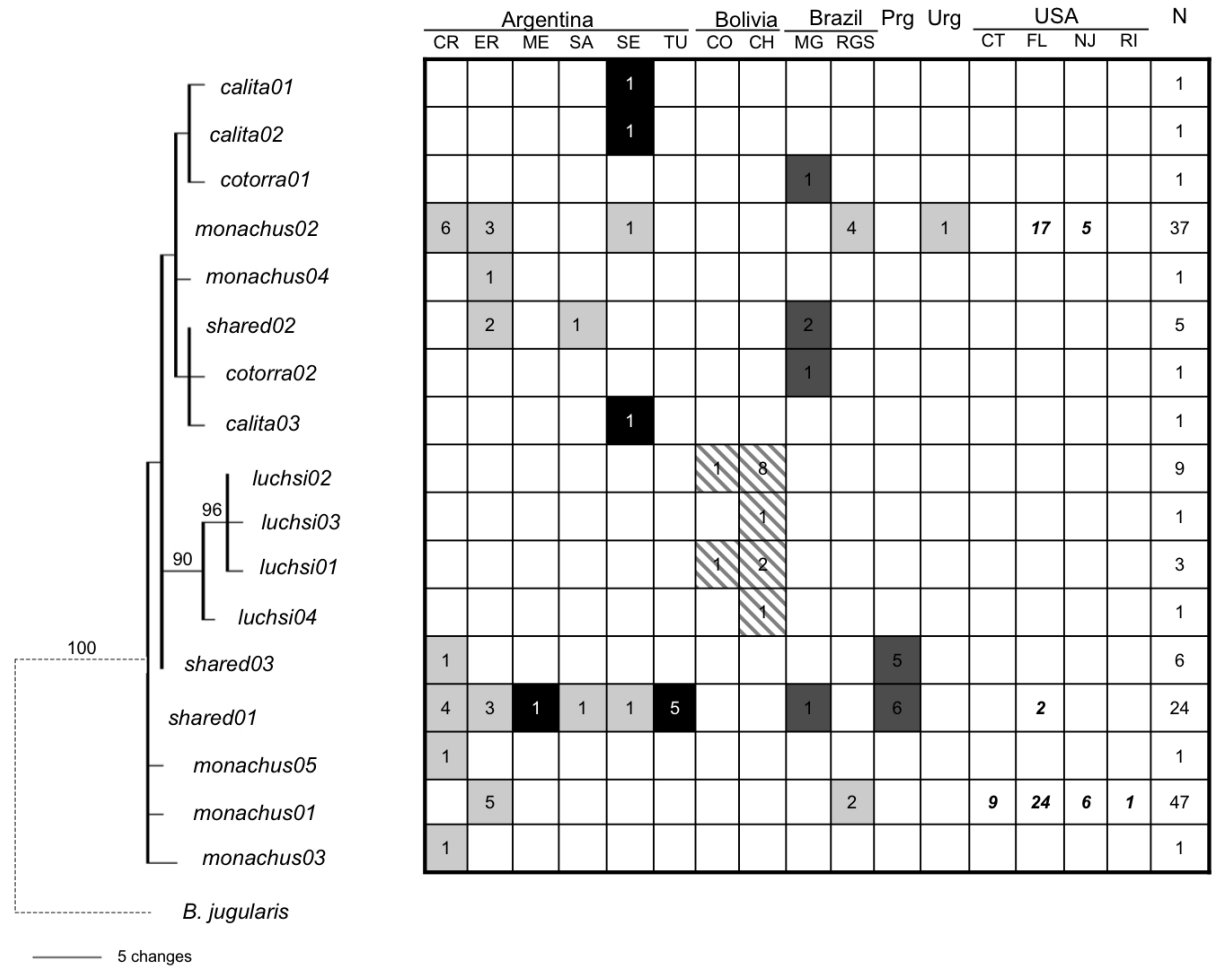
**Bayesian haplotype tree depicting relationships among sampled *Myiopsitta monachus *haplotypes relative to their geographic and taxonomic distributions.** The names of each haplotype are as in Figure 2. Bayesian posterior probabilities (> 50%) are indicated above the branches. Each column in the associated table is a locality sorted by country with abbreviations following Table 1. Each row is a haplotype according to its placement in the tree on the left; the number of individuals at that sampling locality exhibiting that particular haplotype is indicated in each cell. Shading represents the subspecies designation for the distribution of haplotypes according to Figure 1. Bolded italicized numbers indicate the distribution of individuals collected in the naturalized range in the United States. Total number of sampled individuals exhibiting each haplotype (N) is denoted in the last column. For illustration purposes, accurate branch lengths leading to the outgroup are not shown (indicated by dashed line).

A single haplotype network was reconstructed within which all haplotypes had a 95% probability of being parsimoniously connected (Figure 2). Overall, the network was characterized by reticulation and little structure to the recovered relationships (Figure 2). The only distinct clustering was of the four *M. m. luchsi *haplotypes sampled in Bolivia, which were three to four steps different than the nearest *M. m. monachus *or *M. m. cotorra *haplotypes (Figure 2). The remaining haplotypes constituted a mixed assemblage, exhibiting neither geographic structure nor clustering patterns consistent with currently described subspecies boundaries. Results of a Bayesian phylogenetic analysis mirrored those of the haplotype network, reconstructing a well-supported (posterior probability = 90; Figure 3), monophyletic *M. m. luchs*i with the remaining three subspecies forming a paraphyletic assemblage.

### Origin of naturalized populations

Three haplotypes were recovered from the 64 individuals sampled in populations in the eastern United States, all of which were identical to haplotypes detected in the native range of *M. monachus*. The most common haplotype, detected in the naturalized range at a frequency of 0.63, was one previously found unique to *M. m. monachus *(*monachus01*; Figure 3). Initially sampled in Entre Rios, Argentina and Rio Grande do Sul, Brazil, this haplotype was fixed in the Bridgeport, CT (n = 9) and Kent County, RI (n = 1) samplings and was likewise detected at high frequencies in the Miami, FL (0.56) and Edgewater, NJ (0.55) populations. A second high frequency haplotype unique to the *M. m. monachus *subspecies (*monachus02*; Figure 3) was found in the Miami, FL (0.40) and Edgewater, NJ (0.45) populations. In the native range, the *monachus02 *haplotype was recovered over a wide geographic area, found in Rio Grande do Sul, Brazil, Soriano, Uruguay, and throughout sampling localities in northern and central Argentina (Figure 3). Lastly, a haplotype shared by *M. m. monachus*, *M. m. calita *and *M. m. cotorra *in the native range (shared01; Figure 3) was also found at very low frequency in the Miami, FL population (0.03).

## Discussion and conclusion

In this study we employed extensive geographic sampling and historical DNA analysis to describe patterns of genetic variation among subspecies of the invasive monk parakeet in its native range, assess current taxonomic designations, and infer the source(s) of introduced populations in the United States.

### *Myiopsitta monachus* taxonomy

The biological relevance of subspecies has been widely debated since the 1950s [39]. Ernst Mayr, who wrote the most influential book on speciation analysis [40], also grappled with the concept of subspecies. Although in early writings he clearly assigned evolutionary status to subspecies [40], later work directly acknowledged the subjectivity associated with this level of taxonomic classification, explicitly stating that subspecies are not units of evolution [41]. Continentally distributed avian subspecies are a prime example, with a recent survey finding that 97% lack the population genetic structure indicative of historically independent units [42].

We share the view of many that an accurate taxonomy should reflect evolutionary history. The phylogenetic species concept (PSC) offers such an approach, directly linking patterns of evolution with species status [43]. Under the PSC sensu Cracraft [43], a species is the smallest diagnosable cluster of individual organisms within which there is a parental pattern of ancestry and descent. Applied to the monk parakeets, three (*M. m. calita*, *M. m. cotorra*, *M. m. monachus*) of four *M. monachus *subspecies lacked diagnostic character support and violated a criterion of monophyly. The absence of genetic distinctiveness of these three taxa reflects the uncertainty surrounding the differences in size and plumage characteristics upon which they were initially described [11,13]. The lack of discrete morphological character differences in tandem with the results presented here suggest that *M. m. calita, M. m. cotorra *and *M. m. monachus *are in need of formal taxonomic revision.

In contrast to the uncertainty associated with the descriptions of the other three subspecies, the controversy surrounding *M. m. luchsi *has been related to its relative distinctiveness, and proposed elevation to allospecies status [14]. Restricted to the intermontane valleys in Bolivia, *M. m. luchsi *is morphologically distinct from the other subspecies [11], including *M. m. cotorra*, despite the fact that their known ranges come within 175 km of each other [44]. Moreover, *M. m. luchsi *is altitudinally distributed between 1300–3000 m, in sharp contrast to other monk parakeet taxa, which are routinely found below 1000 m. Another unique characteristic involves the cliff-nesting behavior of *M. m. luchsi*, which contrasts with the colonial, tree-nesting exhibited across the remainder of the range of *M. monachus*. This assorted evidence has been used to elevate *M. luchsi *to allospecies status, forming a superspecies with the remaining taxa of *M. monachus *[14]. Although this taxonomic revision is not generally recognized, the results of the current study further highlight the uniqueness of this taxon. In addition to displaying between three and five diagnostic molecular characters relative to *M. m. calita*, *M. m. cotorra*, and *M. m. monachus *(Table 2), the Bolivian *luchsi *formed a well-supported, monophyletic group based on the mtDNA control region sequence data (Figure 3). Collectively, the morphological, behavioral and genetic data support *M. luchsi *as a distinct, phylogenetic species [32,43] and suggest that a formal taxonomic revision is in order.

### Origin of North American populations

Over the past 35 years, monk parakeets have been recorded on U.S. Christmas Bird Counts in 14 states: Connecticut, Delaware, Florida, Georgia, Illinois, Massachusetts, Nebraska, New Jersey, New York, Ohio, Oregon, Pennsylvania, Texas, and Washington D.C./Virginia [13]. Other states where monk parakeet nesting has been observed include Alabama [45], California [46], Louisiana [18], North Carolina [47], South Carolina [48], and Rhode Island [49]. The United States Fish and Wildlife Service conducted an eradication campaign from 1970 to 1975 that effectively eliminated populations in California and reduced the naturalized range of monk parakeets in the U.S. to seven localities in five states [50]. Since 1975, *M. monachus *populations in the U.S. grew exponentially and spread throughout the country to its present distribution [19]. Currently, two of the largest naturalized populations of *M. monachus *reside in Florida and southern Connecticut. Both appear to be expanding in size and geographic distribution. The Florida population, in particular, continues to increase at an exponential rate, with a recent study by Pruett-Jones et al. [51] estimating a statewide population size of 18,025 to 32,044.

Despite multiple introductions and the widespread distribution of *M. monachus *in the U.S., we detected a low level of haplotype diversity across four sampling localities in Connecticut, Florida, New Jersey and Rhode Island. Only three different haplotypes were recovered, the most common of which (*monachus01*; 0.63) was found in all four localities and was identical to a haplotype sampled from *M. m. monachus *in a localized area in eastern Argentina in Entre Rios to Rio Grande do Sul, Brazil on the Uruguayan border. The other high-frequency haplotype in the naturalized range (*monachus02*; 0.34) was also specific to *M. m. monachus *and was likewise sampled in Rio Grande do Sul, Brazil, Soriano, Uruguay and a number of localities in central and northern Argentina. These results are consistent with preliminary morphometric analyses (M. Avery, unpublished) as well as trapping records that indicate that the vast majority of birds captured for the pet trade were *M. m. monachus *exported from eastern Argentina and Uruguay [13]. The concordance between the trapping records and our genetic results support the idea that the invasion of monk parakeets has been facilitated, at least initially, by their widespread presence in the international pet bird trade. As a likely source of large and repeated release events, the international trade in parrots may have historically exerted significant propagule pressure, generally a key determinant of invasion success in birds and other taxa [52,53]. Nuclear data and additional geographical sampling may provide important sources of historical information for further testing this hypothesis.

The Wild Bird Conservation Act of 1992 prohibits the importation of monk parakeets into the United States, reducing the chances of future introductions of wild-caught individuals [54]. However, monk parakeets, known as Quaker parakeets in the pet trade, remain one of the most popular cage birds and are widely bred and sold by aviculturists in the U.S. At the state level, local laws vary, with some states banning the possession of monk parakeets while others placing no restrictions on them. Nevertheless, this domestic trade in monk parakeets remains the most likely source of introductions into states not currently reporting self-sustaining breeding populations [22].

By and large, it is likely that non-native populations of monk parakeets will continue to grow. One reason is that the popularity of monk parakeets has in recent years extended to introduced populations. Efforts to remove birds and nests from electric utility structures in Connecticut, Illinois, Florida, Washington and New Jersey have often met with substantial resistance by a vocal subset of the local communities. In addition, current control strategies have not effectively prevented the establishment and continued growth of naturalized populations in the U.S. and Western Europe, most notably in Spain [17]. Unlike other psittacines, monk parakeets are not constrained by the availability of nesting cavities. Rather, they construct nests of sticks and branches and they tend to select man-made structures as nesting substrates [55]. Furthermore, by exploiting feeding opportunities provided by humans, monk parakeets persist in even cold temperate winters [56]. Population growth and expansion seems assured, as impractically large management efforts would be needed to reverse the trend [57]. Consequently, broader understanding of the mechanisms of monk parakeet invasion success and local adaptation constitute important areas for future basic and applied research.

## Authors' contributions

MAR designed the study, collected samples (museum collections, Connecticut and Rhode Island invasive populations), carried out the molecular studies, performed data analyses, and drafted the manuscript. MLA facilitated sample collection in the U.S. populations, aided in interpretation of results, and helped draft the manuscript. TFW participated in the design of the study, aided in interpretation of results, and helped draft the manuscript. All authors read and approved the final manuscript.
